# Iatrogenic post-intubation tracheal rupture treated conservatively without intubation: a case report

**DOI:** 10.1186/1757-1626-1-259

**Published:** 2008-10-22

**Authors:** Bertrand Prunet, Guillaume Lacroix, Yves Asencio, Olivier Cathelinaud, Jean-Philippe Avaro, Philippe Goutorbe

**Affiliations:** 1 Department of Anaesthesia and Intensive Care, Military Teaching Hospital Sainte Anne 83000 Toulon, France; 2 Department of Otorhinolaryngology, Military Teaching HospitalSainte Anne 83000 Toulon, France; 3 Department of ThoracicSurgery, Military Teaching Hospital Laveran 13998 Marseille, France

## Abstract

**Background:**

Tracheal rupture is a rare but life-threatening complication that most commonly occurrs after blunt trauma to the chest, but which may also complicate tracheal intubation. We report a case of post-intubation tracheal rupture after cataract surgery under general anesthesia treated conservatively.

**Case presentation:**

Four hours after extubation, a 67 year-old woman developed subcutaneous emphysema of the facial, bilateral laterocervical and upper anterior chest. Tracheobronchial fiberendoscopy showed a posterior tracheal transmural rupture 4 cm long located 2.5 cm above the carina that opened in inspiration. The location of the lesion and features of the patient favoured conservative treatment with antibiotic cover. The patient made a full and uncomplicated recovery and was discharged fourteen days after the original injury.

**Conclusion:**

Two therapeutic strategies are currently employed for post-intubation tracheal rupture: a non-surgical strategy for small injuries and a surgical strategy for larger injuries. This case report presented the non-surgical therapeutic strategy of a large tracheal injury.

## Background

Tracheal rupture (TR) most commonly occurs after blunt trauma to the chest, but is also one of the most concerning immediate complications of intubation. Respiratory insufficiency and even death may result from it. We present a case of post-intubation TR after cataract surgery under general anesthesia with orotracheal intubation. The patient was symptomatic only several hours after tracheal intubation, and emergency tracheobronchial fiberendoscopy confirmed the diagnosis. The recovery was uncomplicated following conservative treatment without requirement for intubation. This case report presented the non-surgical therapeutic strategy for a large tracheal injury. Risk factors, clinical, radiological and endoscopic aspects, and different treatments of these tracheal lesions are discussed.

## Case presentation

**Figure 1:**
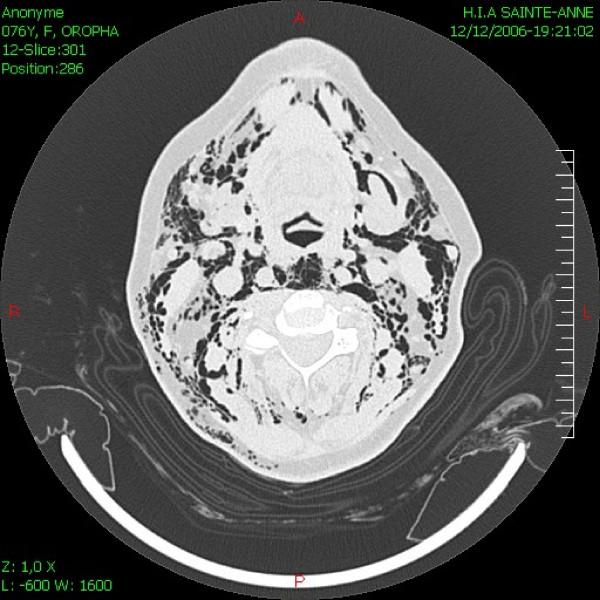
Thoracic CT showing diffuse soft tissues emphysema of the neck.

**Figure 2:**
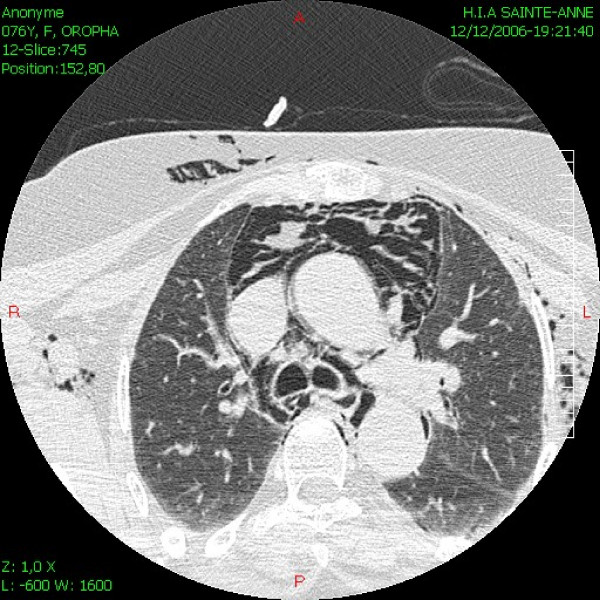
Thoracic CT showing mediastinal emphysema.

**Figure 3:**
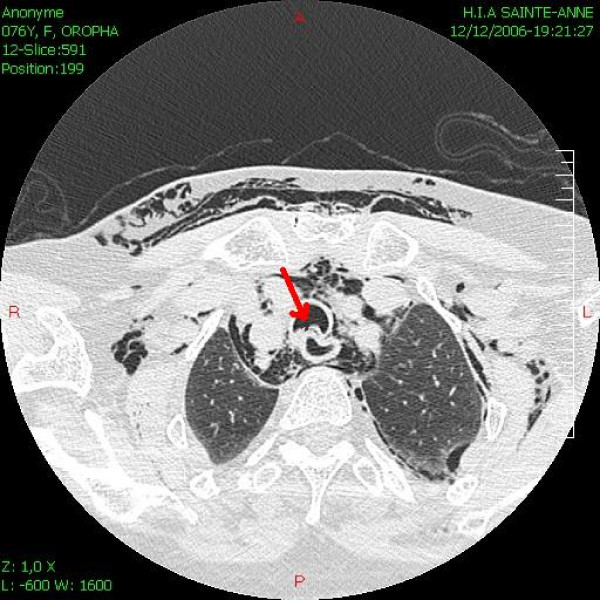
Thoracic CT showing posterior tracheal rupture (Arrow).

**Figure 4:**
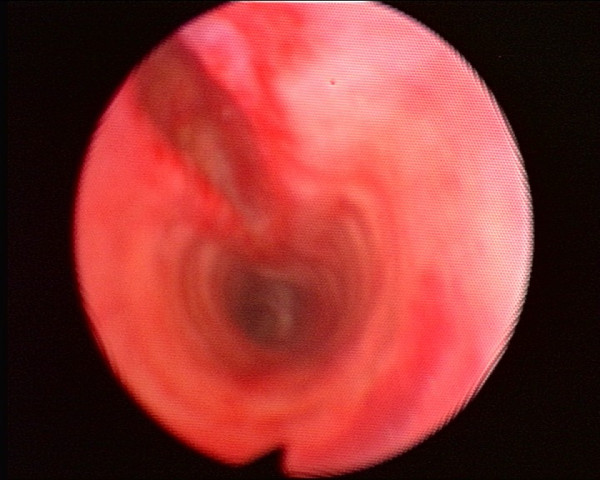
Posterior tracheal rupture.

A 67-year-old woman was scheduled for cataract surgery under general anesthesia. Her only medical history was of arterial hypertension for 25 years that was treated with nifedipine 40 mg/day. The choice of general anesthesia was a request from the patient, as she was too anxious for a topic anesthesia. After induction of anesthesia with propofol 150 mg, sufentanyl 15 mcg and cisatracrium 8 mg, oral intubation was performed without difficulty (Cormack 1) using a 6.5 cuffed preformed orotracheal tube (MALLINCKRODT^®^, Hazelwood, USA) without stylet. The cuff was accidentally overinflated with 20 ml of air. The first obtained cuff pressure was 90 mmHg. The cuff was immediately partially deflated until obtain a pressure of 25 mmHg. The cuff pressure was then monitored during all the surgical procedure. Anesthesia was maintained with sufentanyl and sevoflurane. The entire surgery lasted approximately 40 min. Four hours after extubation, the anesthesiologist was requested because the patient had suddenly developed subcutaneous emphysema of the facial, bilateral laterocervical and upper anterior chest. She had chest pain but no dyspnoea. The chest X-ray did not present any abnormal signs. The results of the arterial blood gas were pH = 7.37 PCO2 = 40.9 mmHg PO2 = 69.2 mmHg HCO3 = 23 mmHg and SpO2 = 93%. A thoracic computed tomography (CT) showed severe and diffuse soft tissues emphysema from the anterior thoracic region to the neck (Figure 1), mediastinal emphysema, both anterior and posterior (Figure 2), and a slightly irregular right lateral outline of the trachea at its medial-distal third (Figure 3). No pneumothorax was observed. Traumatic TR was suspected, and emergency tracheobronchial fiberendoscopy confirmed the diagnosis. It showed a posterior tracheal transmural rupture 4 cm long and located 2.5 cm above the carina, that opened in inspiration (Figure 4 and Additional file 1). Thoracic surgical opinion was that it could be a typically lesion of overinflation tracheal cuff. The location of the lesion and the features of the patient favoured conservative treatment with antibiotic cover (cephalosporin and aminoglycoside) and monitoring in the intensive care unit. Mechanical ventilation, bilateral endobronchial intubation and surgery were available but not necessary. The patient improved, and three days later, the chest CT showed markedly less mediastinal and subcutaneous emphysema. After height days, tracheal fiberendoscopy showed that the lesion was healing. After six more days in a general medical ward, the patient was discharged home in good condition, fourteen days after the initial injury.

## Discussion

Tracheal disruption following intubation is a rare entity [1]. Proposed risk factors for this injury relate to the patient, the operator, the endotracheal tube, the technique of intubation and the anesthesic management [1,2]. Often, this appears to be caused by a combination of these factors [1]. Although in some of the cases, the cause remained unknown [1]. Risk factors relating to the patient include circumstances affecting tracheal anatomy and rigidity that can make the trachea more vulnerable: gender (female), advanced age, short stature, chronic obstructive pulmonary disease and corticosteroid therapy [2]. The prevalence of female patients with iatrogenic tracheal rupture has been previously observed [1,2]. This correlation has led to the assumption that a membranous trachea is less firm in women than in men, but this could be due to the use of an oversized tube rather than for constitutional reasons [3]. Risk factors relating to the operator, the endotracheal tube, the procedure of intubation and the anesthesic management are many [1]: some cases of difficult tracheal intubation that required many intubation attempts could result in iatrogenic tracheal laceration. Nevertheless, often the operators involved were well-trained anesthesiologists and the intubations were reported as "easy" and uneventful. Other causes of iatrogenic tracheal laceration are the choice of an inappropriate tube size, the use of a stylet inside the tube and the stylet not being removed as soon as the tube tip passed the vocal cords, or even the use of a double-lumen tube with direct trauma of the tip or the carina hook [2]. Finally, the overdistension of the cuff of the tracheal tube that acts as a distension force is a frequent cause of TR [1]. In most of the remaining cases, the finding of a clean, sharp-edged longitudinal tear in the posterior membranous part of the trachea, at cervical level (when using a single-lumen tube) or at thoracic level (when using a double-lumen tube), may indicate a cuff-induced rupture [2]. As cuffs are permeable to nitrous oxide and tend to expand during anesthesia, monitoring cuff pressure during surgery is recommended. Normal cuff pressure ranges from 25 to 30 mmHg.

The clinical manifestations of tracheal injury are subcutaneous emphysema, pneumothorax, hemoptysis and respiratory failure [1]. Usually, they appear during surgery or in the immediate postoperative period [2]. Sometimes it does not occur until many hours later [2]. Chest X-ray and chest CT can show soft tissues emphysema, pneumomediastinum, pneumopericardium and/or pneumothorax [2,4]. An emergency bronchoscopy is necessary to establish the diagnosis and to determine the type and extension of the laceration [1,2,5].

Adequate treatment strategy of a post-intubation TR is dependent on the size and the location of the rupture, its clinical presentation, and the overall condition of the patient [1]. Two therapeutic strategies are presently proposed: a surgical or non-surgical approach [3]. Determining which is the best treatment of TR after tracheal intubation is challenging. The current tendency is to decrease invasive surgical treatment for the benefit of conservative management [1,3,6]. Early surgical repair is the preferred treatment for most patients when a transmural tear with a length exceeding 2 cm causes pneumothorax and/or pneumomediastinum and when the lesion is discovered during a thoracic surgical procedure [7]. Conservative treatment may be a viable alternative for some patients [8]: small rents, short lacerations in the upper third of the trachea, especially if they do not involve the entire thickness of the tracheal wall, absence of gross air leak and respiratory embarrassment, or patients who are breathing spontaneously. Conservative treatment can be also used in patients with a large complete longitudinal tracheal rupture, in which old age and critical general condition do not allow surgical intervention due to the life-threatening risk of a major surgical trauma [3]. The practical achievement of conservative treatment can include mechanical ventilation after tracheal or bilateral endobronchial intubation (with the cuff inflated distal to the tear), chest drain, continuous airway humidification, broad-spectrum antibiotics (using a combination of beta-lactam and aminoglycoside because mediastinitis and sepsis can occur in the long term), and chest physiotherapy [3]. In every case, good healing and the absence of stenosis must be remotely verified with tracheal fiberendoscopy one month after the initial injury [3].

## Conclusion

This case report presented an iatrogenic post-intubation tracheal rupture treated conservatively. It illustrates the effectiveness of the non-surgical therapeutic strategy of a large tracheal injury. Selection of treatment for post-intubation TR must remain individualized. But this case illustrates the current tendency to increase conservative therapy in this pathology.

## Competing interests

The authors declare that they have no competing interests.

## Authors' contributions

Each of the authors cared for the patient. BP and PG drafted the manuscript. GL and YA prepared the figures and the video file. Each of the authors has read and approved the manuscript.

## Supplementary Material

Additional file 1 Movie 1. Tracheobronchial fiberendoscopy showing a tracheal rupture Click here for file
